# The hub-and-spoke organization design: an avenue for serving patients well

**DOI:** 10.1186/s12913-017-2341-x

**Published:** 2017-07-11

**Authors:** James K. Elrod, John L. Fortenberry

**Affiliations:** 1 Willis-Knighton Health System, 2600 Greenwood Road, Shreveport, LA 71103 USA; 2 LSU Shreveport, 1 University Place, Shreveport, LA 71115 USA

**Keywords:** Hub-and-spoke, Organization design, Healthcare delivery networks, Medical care

## Abstract

**Background:**

The healthcare industry is characterized by intensive, never-ending change occurring on a multitude of fronts. Success in such tumultuous environments requires healthcare providers to be proficient in myriad areas, including the manner in which they organize and deliver services. Less efficient designs drain precious resources and hamper efforts to deliver the best care possible to patients, making it imperative that optimal pathways are identified and pursued. One particular avenue that offers great potential for serving patients efficiently and effectively is known as the hub-and-spoke organization design.

**Discussion:**

The hub-and-spoke organization design is a model which arranges service delivery assets into a network consisting of an anchor establishment (hub) which offers a full array of services, complemented by secondary establishments (spokes) which offer more limited service arrays, routing patients needing more intensive services to the hub for treatment. Hub-and-spoke networks afford many benefits for healthcare providers, but in order to capitalize fully, proper assembly is required. To advance awareness, knowledge, and use of the hub-and-spoke organization design, this article profiles Willis-Knighton Health System’s service delivery network which has utilized the model for over three decades. Among other things, the hub-and-spoke organization design is defined, benefits are stipulated, and applications are discussed, permitting healthcare providers essential insights for the establishment and operation of these networks.

**Conclusions:**

The change-rich nature of the healthcare industry places a premium on incorporating advancements that permit health and medical providers to operate as optimally as possible. The hub-and-spoke organization design represents an option that, when deployed correctly, can greatly assist healthcare establishments in their quests to serve patients well.

## Background

Health and medical providers operate in an industry characterized by perpetual change, the result of a convergence of multiple influences from both within and outside of their given establishments. The healthcare industry, in and of itself, is immensely complex, and its immersion in and exposure to the greater political, economic, social, and technological environment only adds to associated complexities [1, 2].

Successfully navigating such tumultuous environments requires that healthcare providers be proficient in myriad areas, including the manner in which they organize and deliver services. Less efficient designs drain precious resources and hamper efforts to provide the best care possible to patients, making it imperative that optimal pathways are identified and pursued. One particular avenue that offers great potential for serving patients well is known as the hub-and-spoke organization design [3, 4].

Through strategic centralization of the most advanced medical services at a single site and distribution of basic services via secondary sites, the hub-and-spoke model affords unique opportunities to maximize efficiencies and effectiveness. A well-designed hub-and-spoke network satisfies patient care needs fully, yet does so in a manner that fosters resource conservation, return on investment, service excellence, and enhanced market coverage [4–7]. Benefits abound, but in order to capitalize fully on the hub-and-spoke organization design, healthcare providers must assemble their service delivery networks with great care and attention.

To advance awareness, knowledge, and use of the hub-and-spoke organization design, this article profiles Willis-Knighton Health System’s service delivery network which has utilized the model for over three decades. Among other things, the hub-and-spoke organization design is defined, benefits are stipulated, and applications are discussed, permitting healthcare providers essential insights for the establishment and operation of these networks.

### Definition

Formally defined, the hub-and-spoke organization design is a model which arranges service delivery assets into a network consisting of an anchor establishment (hub) which offers a full array of services, complemented by secondary establishments (spokes) which offer more limited service arrays, routing patients needing more intensive services to the hub for treatment [3, 7]. The hub-and-spoke model yields a healthcare network consisting of a main campus and one or more satellite campuses. It is much more efficient than organization designs which replicate operations across multiple sites [5, 7, 8]. Hub-and-spoke networks are highly scalable, with satellites being added as needed or desired [6, 7]. When geographic distance makes satellite-to-hub access impractical, an additional hub can be created, yielding a multi-hub network [4, 5, 9].

The particular manner of centralization varies from institution to institution, depending on the service array provided and size of market addressed, but a common approach is as follows: Complex medical services, especially those that are technology and skills intensive, are centralized at the main campus or hub, as are services that support care delivery and lend themselves to centralization, such as human resource management, marketing, and related operations. Basic healthcare services are broadly distributed across the network, permitting the bulk of healthcare needs of the populace to be addressed locally. Only when complexities emerge that require care falling outside of the scope of services provided at satellite facilities are patients routed to the main campus or hub for treatment [3, 4, 7].

The hub-and-spoke model has origins in the transportation industry. It is perhaps best known in broad society for its use by air carriers, which due to resource scarcity and intensive demands for profits, operate these networks to accomplish more with less [10, 11]. The model has been adopted by and used successfully in many other industries, including retailing, education, and healthcare [12–14].

Strategic centralization is the key which unlocks the many benefits of the hub-and-spoke organization design [3–5, 7]. Informed decisions regarding service distribution are essential, with the goal being to find an optimal balance between operations efficiency and quick, convenient access by patients to associated healthcare services. This is something that only healthcare providers themselves can decide, as service arrays, the markets in which they are offered, and populations served are unique to each entity. However, with firsthand familiarity of an operational hub-and-spoke network, knowledge and awareness of this organization design comes to life, making construction of custom networks possible for most any interested healthcare provider. Willis-Knighton Health System’s service delivery network makes use of the hub-and-spoke model, offering an excellent opportunity to gain an understanding of this particular organization design’s attributes and operation.

### Willis-Knighton Health System and its hub-and-spoke network

Headquartered in Shreveport, Louisiana, Willis-Knighton Health System is a nongovernmental, not-for-profit healthcare provider delivering comprehensive health and wellness services through multiple hospitals, numerous general and specialty medical clinics, an all-inclusive retirement community, and more. The system holds market leadership in its served region, centered in the heart of an area known as the Ark-La-Tex, where the states of Arkansas, Louisiana, and Texas converge.

Willis-Knighton Health System’s origins date to 1924 with the establishment of Tri-State Sanitarium, founded to address the healthcare needs of the burgeoning population of west Shreveport. Sold in 1929 to Drs. James Willis and Joseph Knighton, the establishment continued operations and, in 1952, it was renamed in honor of Drs. Willis and Knighton.

For the first several decades of its existence, the establishment played an important but relatively small role in delivering the region’s healthcare. In the 1970s, however, Willis-Knighton Health System embarked on a detailed growth campaign to expand its footprint beyond west Shreveport. With funding to support growth initiatives being in short supply, executives were forced to economize, with this creating a culture of efficiency, something that characterizes Willis-Knighton Health System to this day.

Through many years of effort and innovation, growth was realized. By the early 1980s, Willis-Knighton Health System’s sole hospital, located on Greenwood Road in the shadow of downtown Shreveport, had witnessed an exceptional increase in patient volume and loyalty was at an all-time high. These successes afforded opportunities to explore avenues for expansion. In doing so, executives noticed that the population of south Shreveport was expanding greatly but no hospital was serving the area. This represented a natural pathway to increase Willis-Knighton Health System’s footprint and build patient volume in a new and growing area of the city. South Shreveport’s residential developments were increasing rapidly, but its commercial infrastructure was relatively undeveloped, leading executives to pursue a new construction pathway for realization of the proposed hospital campus.

Although south Shreveport presented the region’s most attractive growth prospects, Willis-Knighton Health System did not want to abandon its original campus. Prospects for growth remained good in and around the west Shreveport and downtown areas of the city. Further, a departure would effectively have stranded patients in that particular region, a practice that the system has always rejected as a not-for-profit, charitable enterprise. Departure also would have opened the door for competitors to easily enter the west Shreveport/downtown marketplace and capture that market share for themselves. As such, executives decided to operate two hospital campuses, the original one in west Shreveport and a new one in south Shreveport. Land in south Shreveport was acquired and planning ensued.

Among myriad decisions, executives needed to determine the manner in which healthcare delivery relationships between the two establishments would operate. Organization designs which essentially replicated service arrays at each site were popular expansion models at the time, but this pathway went against Willis-Knighton Health System’s culture of efficiency. Seeking a more practical approach, various models were explored and the hub-and-spoke organization design was selected due to its reputation for efficient and effective service delivery.

Service array and operations protocols for the proposed facility were determined, architectural designs were developed and approved, and construction began. In 1983, Willis-Knighton Health System revealed WK South, its first satellite hospital, beginning its journey as a multicampus health system. Through this experience, the efficiency and value of the hub-and-spoke organization design was confirmed, prompting its continued use [15].

**Fig. 1:**
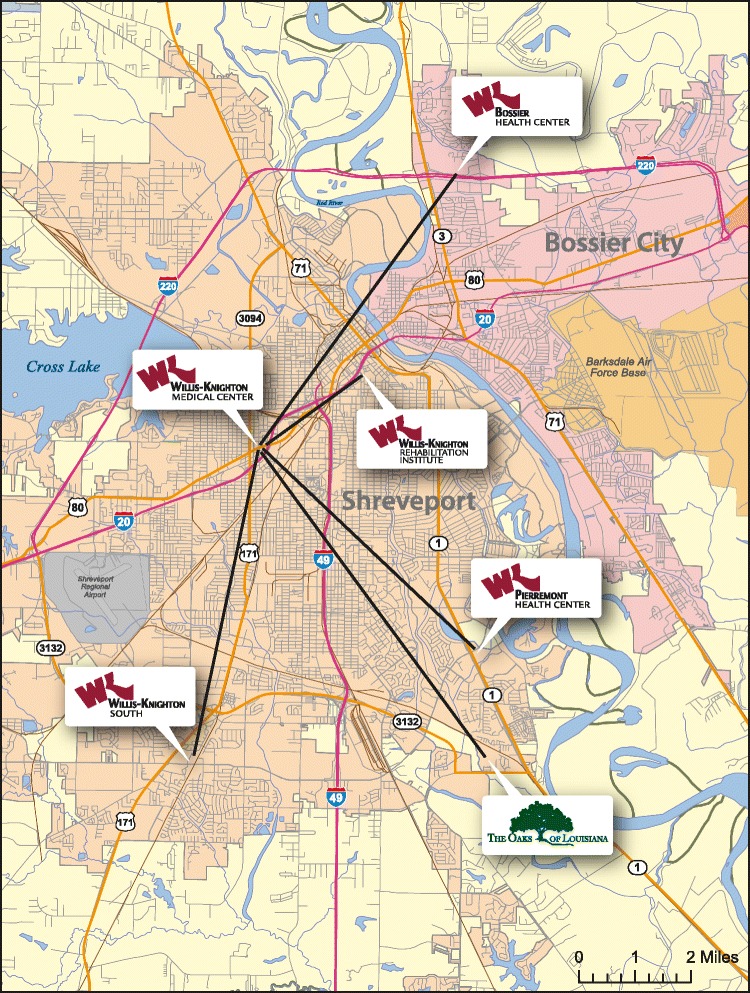
A map presenting Willis-Knighton Health System’s hub-and-spoke network. Copyright © 2017 Willis-Knighton Health System. Used with permission. Willis-Knighton Health System’s hub-and-spoke network consists of one hub, Willis-Knighton Medical Center, and five primary spokes: WK South, WK Bossier Health Center, WK Pierremont Health Center, WK Rehabilitation Institute, and The Oaks of Louisiana. Numerous general and specialty medical clinics, located throughout the region, also serve as spokes linked to the main campus hub

Willis-Knighton Health System now operates 1290 licensed beds across five hospital campuses and one retirement community. The main campus, Willis-Knighton Medical Center, serves as the hub for each of the satellite campuses, as illustrated in Fig. 1. The system’s numerous general and specialty medical clinics, located throughout the region, also serve as spokes linked to the main campus hub. Willis-Knighton Health System’s five hospital campuses and retirement community, their establishment dates, and current licensed bed sizes (as of March 2017) are as follows.

- WK Medical Center (hub, est. 1924): 499 beds
- WK South (spoke, est. 1983): 152 beds
- WK Bossier Health Center (spoke, est. 1996): 166 beds
- WK Pierremont Health Center (spoke, est. 1999): 200 beds
- WK Rehabilitation Institute (spoke, est. 2017): 65 beds
- The Oaks of Louisiana (spoke, est. 2007): 208 beds

Detailed service arrays provided at each location can be accessed by visiting Willis-Knighton Health System’s website (http://www.wkhs.com). With the exception of The Oaks of Louisiana, which involved the acquisition of a small long term care campus that subsequently was developed into a comprehensive retirement community, the spokes in Willis-Knighton Health System’s hub-and-spoke network were established entirely by the system. System growth experienced over the decades can be credited in large part to the hub-and-spoke model which permitted the best use of resources to achieve growth ambitions and serve increasing populations of patients well.

### Benefits

With over three decades of delivering healthcare services using the hub-and-spoke organization design, Willis-Knighton Health System can confirm a range of benefits afforded by the model. These benefits, which mirror observations expressed in the literature, are summarized in Table 1 and explained as follows.

**Table 1:** Benefits and risks associated with the hub-and-spoke organization design

Benefits	
a. Consistency across operations	
b. Increased efficiencies	
c. Enhanced quality	
d. Enhanced market coverage	
e. Improved agility	
Risks	
a. Congestion at hubs	
b. Overextension of spokes	
c. Staff dissatisfaction at spokes	
d. Transportation disruptions	

#### Consistency across operations

The hub-and-spoke organization design affords significant command and control across the associated network of healthcare establishments. Policy directives are issued system wide by the main campus or hub, with administrators at satellite facilities being responsible for implementation in their assigned establishments. This yields consistency across operations, something that would be much more difficult to achieve in networks consisting of semi-independent or independent facilities [7, 9].

The hub-and-spoke model effectively creates a hierarchical organization with authority extending from the hub outward to the spokes. This yields a traditional chain of command similar to that used by military organizations. Perhaps most importantly, from a patient care perspective, the uniformity associated with this particular organization design affords a consistent patient experience across facilities [9].

Courtesy of the hub-and-spoke organization design, despite operating multiple campuses, there really is only one Willis-Knighton Health System and it’s composed of the entire collection of establishments. Patients with experience at one Willis-Knighton Health System campus will find similar experiences at any of the system’s other campuses. Each spoke is managed by an individual holding the title of Administrator and Vice President who reports directly to the hub-based President and Chief Executive Officer. Medical staff members are credentialed for service system wide; they are coordinated by a hub-based Vice President. A single governing board is responsible for system oversight. Central command afforded by the hub-and-spoke network allows each of Willis-Knighton Health System’s campuses to quickly and collectively adapt to environmental changes as directives to modify practices come from a single source.

#### Increased efficiencies

By centralizing the most advanced medical technologies and skill sets at the main campus or hub of the hub-and-spoke network and routing all patients needing these services to this central site, efficiencies are achieved. Notably, this eliminates the costly duplication of services, increases return on investment, and bolsters economies of scale [4–7]. Evidence also suggests that the cost of care delivered by hub-and-spoke networks is reduced, benefiting patients, insurers, and society at large [16–18].

Willis-Knighton Health System follows the practice of centralization without fail, concentrating leading technologies at its hub. These especially expensive technologies, in fact, are not permitted at Willis-Knighton Health System’s spoke facilities unless patient demand is sufficient at these satellite locations to justify the expenditures. Further, medical staffing, human resources, marketing, materials management, and finance operations are based at the main campus and serve the entire system from that location. These actions yield tremendous efficiencies which positively impact financial performance and afford opportunities to reinvest savings in pursuits that otherwise might not be possible.

The efficiencies generated by the hub-and-spoke organization design have, among other things, permitted Willis-Knighton Health System to acquire some of the most sophisticated medical technologies available, such as proton therapy for the treatment of cancer, Visualase MRI-guided laser ablation technology for treating brain tumors, and much more. The resulting savings also facilitates charity care initiatives, permitting the system to provide healthcare services to a significant percentage of the region’s uninsured population.

#### Enhanced quality

Due to its unique structure, pooling resources and expertise strategically, the hub-and-spoke organization design offers opportunities to realize quality enhancements [4], with findings in the literature supplying evidence of the model’s ability to deliver improved outcomes [19–22]. In many regards, the hub of the hub-and-spoke network effectively becomes a system-wide center of excellence, as it contains many like attributes, including centralization which concentrates resources at single sites and bolsters patient volume, fostering quality [23–25].

Willis-Knighton Health System’s experiences confirm the guidance supplied in the literature pertaining to the quality impact of the hub-and-spoke model. Located at the system hub, its WK Heart and Vascular Institute, for example, houses some of the most sophisticated technologies available and it is staffed by the most experienced cardiovascular team in the region. The concentration of leading resources, together with significant volume resulting from system-wide referrals (597 cardiovascular surgeries were performed in 2016), has afforded a level of quality that would be difficult to achieve otherwise. Quality accomplishments and outcomes experienced on the cardiovascular surgery front also have been observed across other clinical fronts in the system, demonstrating value and warranting continued use of the hub-and-spoke organization design.

#### Enhanced market coverage

The hub-and-spoke organization design facilitates expansion initiatives, courtesy of the reduced resource requirements necessary at the satellite facility level. Since service arrays at satellites are more limited in scope, less investment is needed to establish new locations, something that also reduces the risk associated with expanding into new markets. As such, the hub-and-spoke model offers significant opportunities to bolster market share and improve access to care [4–7]. This very benefit compelled and permitted Willis-Knighton Health System to begin development of what will become its sixth hospital, WK Palmetto Center, which will be located in the fast-growing town of Benton, Louisiana, north of Bossier City.

The hub-and-spoke model, in fact, has been receiving increasing attention of late as a potential solution to many population health challenges [5, 26]. Logically, resolution of historic and ongoing access to care dilemmas should be easier if expansion efforts can be accommodated at less cost and reduced risk, as made possible by well-planned hub-and-spoke networks. Willis-Knighton Health System’s own outreach initiatives into medically underserved communities have increased in depth and breadth largely due to the efficiencies and abilities of its hub-and-spoke network.

#### Improved agility

The synergies associated with operational consistency, efficiencies, and enhanced market coverage combine to yield an especially agile healthcare organization. Change initiation is expedited and enhanced through the central administrative structure and less costly, reduced-risk expansion opportunities foster expeditious decisions and resulting actions [3, 4, 7].

The hub-and-spoke model also enhances an institution’s ability to adapt as markets evolve. When attractive markets emerge, cost efficient satellites facilitate nimble market entry. Due in large part to the efficiencies of the hub-and-spoke organization design, Willis-Knighton Health System was able to address each market in which it placed a satellite hospital more quickly than did its competitors, a trend which continues with its newest campus development initiative in Benton, Louisiana.

Conversely, in situations where particular markets served by satellites are declining, withdrawal can occur swiftly. Those resources can then be redirected to other establishments in the network or they perhaps can be used to build new establishments in markets with greater potential. Interestingly, this particular facet can also benefit hubs in cases where market decline or failure is experienced at the main campus. In such cases, satellites present opportunities to transfer resources and reestablish the hub in a more promising market.

Willis-Knighton Health System has been fortunate to have never experienced the need to close any of its campuses, but prudent healthcare executives must always be mindful that circumstances beyond their control can force painful decisions to be made in order to protect institutional viability. In the event of market downturns beyond levels of tolerance, the hub-and-spoke model offers perhaps the best prospects for ensuring continuity in the face of one or more closures. Flexibility in such situations is an asset and the hub-and-spoke model delivers just that.

### Risks

While the hub-and-spoke organization design offers a wealth of benefits, this particular approach to organizing the delivery of healthcare services does carry some risk, with potential trouble spots being summarized in Table 1 and explained below. Fortunately, steps can be taken to reduce or eliminate these negative occurrences.

#### Congestion at hubs

Since patient traffic is routed from one or more spokes to the hub, the potential naturally exists for congestion to occur at the main campus [27, 28]. With proper planning and action, however, such occurrences can be minimized or possibly eliminated altogether. Quite simply, steps must be taken to ensure that system-wide demand directed toward the hub can be accommodated without hardship or delay. This requires addressing several fronts, including parking access and availability, scheduling practices, space requirements, technology availability and access, staff coverage, and so on.

Once again, centralization delivers benefits, in this case, pertaining to the supply of intelligence that will aid in devising plans to accommodate patient demand. The main campus, courtesy of the hub-and-spoke design, serves as the system-wide data repository, housing the electronic health records system, among other things, for the entire network. This central structure permits historic and real-time access to patient volume and flow information, providing an invaluable planning resource. Also, since a nexus of top expertise and equipment exists at the hub, scaling capacity to meet demand is much easier than starting anew.

All the more effort will be needed in this area in cases where new satellites are constructed or otherwise acquired, as this will require actions to ensure that the hub has the capacity to address new volume. Willis-Knighton Health System engages in intensive planning beginning early in the development phases of satellite campuses through to construction and commercialization to ensure adequacy of resources needed to accommodate demand and ensure positive patient experiences. Proactive planning can greatly reduce or eliminate the potential for congestion, ensuring convenient and expeditious access to patient care.

#### Overextension of spokes

Spoke overextension—placing satellites too distant from the hub to permit adequate service delivery—must be avoided. This invariably will cause service failures that will tarnish the reputations of both the satellite and the main campus [15]. As noted earlier, strategic decisions are required to strike a balance between the desires of institutions for operational efficiency and the desires of patients for receipt of services in close proximity to their homes.

Optimal decisions can only be made by investigating the particular scenario, with attention being directed toward the type of service, type of patient, distance from hub, state of and access to transportation, and so on in order to determine the most prudent pathways. If transit times between the hub and satellite are too great to permit effective care delivery, then efforts should be directed toward establishing a hub in this remotely-located market to ensure continuity of care and allow future satellite growth in the region.

Willis-Knighton Health System’s satellite campuses are located in reasonably close proximity to the hub, permitting quick access via personal transportation. The network also benefits from the system’s highly-developed ground and air patient transportation program which ensures the most expeditious spoke-to-hub access possible in life-threatening situations. Expansion into any market begins with a community needs assessment which includes transit time and related analyses, offering helpful guidance to executives as they go about determining the adequacy of spoke-to-hub access to ensure that overextension is avoided.

#### Staff dissatisfaction at spokes

Since authority runs from the hub outward to the spokes, satellite facilities operate, by design, under the auspices of the main campus. This sets the stage for at least some staff members at spoke facilities to be discontent, resenting the lack of autonomy to operate as they see fit within their facilities [15]. They might, for example, wish to schedule work differently, determine services that will and will not be offered, devise their own advertising campaigns, or develop site-specific personnel policies. Such actions undermine command and control structures and over time will create patient experiences that are not uniform across the system.

Outside of ensuring clear communication between and among all establishments in the hub-and-spoke network, the threat of staff dissent can be reduced by making certain that satellite facilities are led by capable administrators who embrace the system’s corporate culture and appropriately guide their subordinates. This, of course, places a premium on ensuring that a constructive culture exists within the institution and that it is conveyed successfully throughout the network. Offering an informative employee orientation and periodic training opportunities which include details regarding the mission, vision, and values of the organization can further bolster team mindsets that minimize strife.

Willis-Knighton Health System’s belief in establishing a positive, productive organizational culture and providing continual learning opportunities prompted it to develop the WK Innovation Center, a site specifically designed for the provision of system-wide education initiatives. Personnel from across the system visit this particular location for their initial onboarding and they return periodically for a wealth of training opportunities throughout their careers with Willis-Knighton Health System. Such initiatives have greatly facilitated the system’s efforts to foster cohesion and collaboration across the network.

Ultimately, matters of discontent are highly addressable with numerous remedies being available. Proactive steps can help to ensure the presence of positive, collaborative work environments across the hub-and-spoke network, providing mutual benefits that permit the delivery of outstanding care to patients.

#### Transportation disruptions

Hub-and-spoke healthcare delivery networks, by their very nature, are reliant on transportation systems, with linkages between hubs and satellites being critical for patients to realize the entire continuum of care offered by providers [27, 29, 30]. Common options for satellite-to-hub access include personal transportation, patient transport vans, and emergency vehicles and aircraft. These modes of transportation, of course, rely on transit networks and their accessibility. Municipal road construction projects, bridge closures, bad weather, and the like can create significant service delivery obstacles, as can vehicle breakdowns, traffic accidents, insufficient patient transportation systems, and so forth. Some of these are beyond the control of healthcare institutions (e.g., severe weather which grounds air ambulances), but others (e.g., assembly of proper patient transportation systems) can be managed with concerted effort and attention.

Willis-Knighton Health System’s patient transportation division provides ground and air transit services which are delivered by personnel dedicated to ensuring that patient access needs are addressed throughout the network. Proper servicing of vehicles, aircraft, and equipment helps to ensure readiness and effective operation. Safety training fosters good habits that reduce the potential for accidents. Area roadways are in good order and, in the event of road or bridge closures, alternate routes are readily available. Willis-Knighton Health System has never experienced any significant transportation disruption that hampered the operation of its hub-and-spoke network. Still, providers making use of this model of organization design must not neglect their reliance on transportation systems and do their part to reduce the potential for failures to ensure top institutional performance.

### Operationalization

Formulating a hub-and-spoke network ultimately requires that healthcare providers acquire an understanding of the tenets of the associated organization design and then direct careful thoughts and actions toward structuring healthcare delivery, accordingly. Similar to the pathway followed by Willis-Knighton Health System to realize its own hub-and-spoke network, a growth-minded healthcare institution would simply designate a hub of operation, often its current establishment, and plan to concentrate the bulk of attributes and abilities at this main campus. Growth then is pursued by building or otherwise acquiring spokes, with these satellite facilities offering a limited selection of healthcare services, routing cases requiring more intensive medical interventions to the main campus or hub for treatment.

Significant effort must be directed toward structuring relationships between and among network establishments. Service arrays, reporting relationships, and other operational protocols should be defined very clearly. Further, healthcare providers must take great care to ensure that operations are designed and managed effectively to prevent hub congestion, avoid spoke overextensions, facilitate system-wide staff cohesion, and foster expeditious access via productive transportation systems. Notably, hub-and-spoke networks are highly adaptable, permitting most any enterprising healthcare establishment, regardless of its size or mission, to make use of the model to enjoy its many benefits. Realization ultimately is a matter of properly designing, structuring, and maintaining organizational relationships.

## Conclusions

The hub-and-spoke organization design has greatly facilitated Willis-Knighton Health System’s efforts to operate in a fiscally responsible and highly effective manner. By embracing this particular method for organizing and delivering healthcare services, economical expansion was permitted which allowed the system’s growth ambitions to turn into achievements. Perhaps most importantly, those residing in northwest Louisiana have been afforded access to an extensive and ever-advancing range of healthcare options, fulfilling Willis-Knighton Health System’s mission “To continuously improve the health and well-being of the people we serve.”

The change-rich nature of the healthcare industry places a premium on incorporating advancements that permit health and medical providers to operate as optimally as possible. As evidenced by the experiences of Willis-Knighton Health System, the hub-and-spoke organization design represents an option that, when deployed correctly, can greatly assist healthcare establishments in their quests to serve patients well.
